# Histone H4 induces platelet ballooning and microparticle release during trauma hemorrhage

**DOI:** 10.1073/pnas.1904978116

**Published:** 2019-08-12

**Authors:** Paul Vulliamy, Scarlett Gillespie, Paul C. Armstrong, Harriet E. Allan, Timothy D. Warner, Karim Brohi

**Affiliations:** ^a^Centre for Trauma Sciences, Blizard Institute, Barts and the London School of Medicine and Dentistry, Queen Mary University of London, E1 2AT, United Kingdom;; ^b^Centre for Immunobiology, Blizard Institute, Barts and the London School of Medicine and Dentistry, Queen Mary University of London, E1 2AT, United Kingdom

**Keywords:** platelets, trauma, coagulopathy, histones, hemorrhage

## Abstract

Trauma hemorrhage is a leading cause of death and disability worldwide. Platelets are fundamental to primary hemostasis, but become profoundly dysfunctional in critically injured patients by an unknown mechanism, contributing to an acute coagulopathy which exacerbates bleeding and increases mortality. The objective of this study was to elucidate the mechanism of platelet dysfunction in critically injured patients. We found that circulating platelets are transformed into procoagulant balloons within minutes of injury, accompanied by the release of large numbers of activated microparticles which coat leukocytes. Ballooning platelets were decorated with histone H4, a damage-associated molecular pattern released in massive quantities after severe injury, and exposure of healthy platelets to histone H4 recapitulated the changes in platelet structure and function observed in trauma patients. This is a report of platelet ballooning in human disease and of a previously unrecognized mechanism by which platelets contribute to the innate response to tissue damage.

## Significance

Membrane ballooning is a fundamental mechanism by which platelets contribute to thrombin generation. However, this process has not previously been described in human disease. In this study, we demonstrated the presence of ballooning procoagulant platelets free in the circulation of critically injured humans, a phenomenon which results in systemic generation of thrombin and contributes to an acute coagulopathy. The surfaces of ballooning platelets were decorated with the damage-associated molecular pattern histone H4, and exposure of healthy platelets to histone caused membrane disruption and recapitulated the phenotypic changes in injured patients. These findings provide a description of platelet ballooning contributing to human disease and identify histone release from injured tissues as a driver of the procoagulant ballooning process.

Platelets are the primary cellular effectors of hemostasis, but become profoundly dysfunctional in critically injured patients (1–3). A global loss of platelet aggregatory function is part of an acute coagulopathy which develops within minutes of injury, exacerbates bleeding, and has a major impact on the risk of multiple-organ failure and mortality (4, 5). The mechanisms underlying trauma-induced platelet dysfunction are unknown, but appear to result from an as yet undefined soluble factor in the plasma of trauma patients (6). Current transfusion protocols use platelet concentrates to support platelet function, but these do not restore platelet responsiveness during active hemorrhage (3, 7). Despite this, platelet transfusions appear to be critical to the survival of trauma patients, but again the mechanisms responsible are unclear (8). As nearly half of the 5 million trauma deaths every year are due to hemorrhage (9–11), understanding the function of platelets in bleeding trauma patients is vital for progress in the field.

The objective of this study was to elucidate the nature of and mechanisms underpinning the phenotypic changes in platelets in critically injured patients. We examined platelets and platelet responses in blood samples taken from trauma patients immediately on arrival at the trauma center. Here we show that reduction in the ability of platelets to aggregate occurs in parallel with an increase in their procoagulant function. Using advanced image analyses, we describe the transformation of platelets into procoagulant balloons, accompanied by release of large numbers of activated microparticles which coat leukocytes. We further show that histone H4, a damage-associated molecular pattern released into the circulation as a result of tissue damage and shock (12), interacts with circulating platelets after trauma and can entirely recapitulate these phenomena through its direct action on platelet membranes. Thus we identify a central pathway responsible for inducing a profound platelet function switch in critically injured patients dependent upon extracellular histones driving platelet ballooning and activated microparticle production.

## Results

### Thrombin Production Is Maintained in Patients with Platelet Dysfunction Despite Procoagulant Factor Loss.

**Fig. 1.**
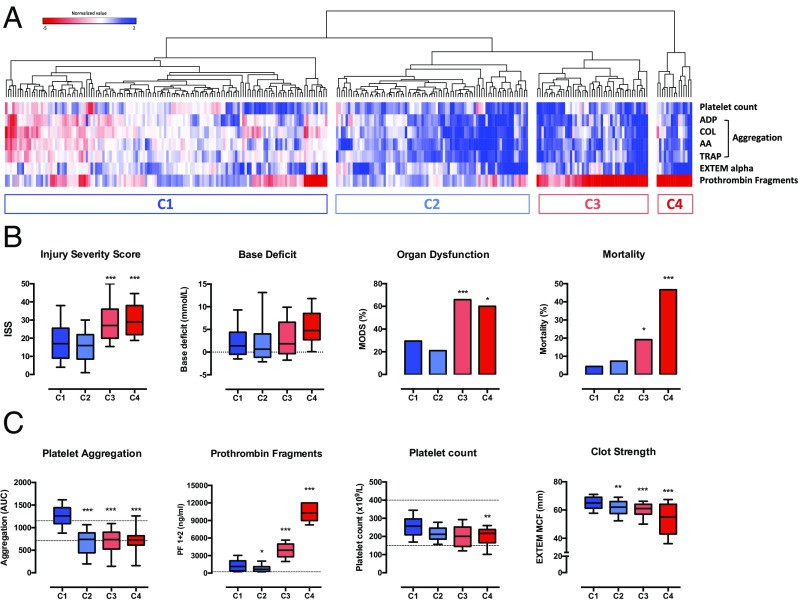
Hierarchical clustering analysis of platelet function parameters in trauma patients. (*A*) Heatmap and dendrogram illustrating 4 major clusters of patients (C1–C4). Each column represents a patient, and each row represents a platelet function parameter. Red cells indicate values which are increased relative to the reference population; blue cells indicate values which are reduced relative to the reference population. (*B*) Injury characteristics and outcomes in the 4 clusters. (*C*) Coagulation and platelet function profile in the 4 clusters. Box plots depict median, interquartile range and 10th–90th percentiles. Dashed lines denote normal range. *P < 0.05 **P < 0.01 ***P < 0.001 vs. cluster 1, 1-way ANOVA with Tukey’s posttest for multiple comparisons.

We performed impedance aggregometry, thromboelastometry, and measured circulating prothrombin fragments in a cohort of 279 injured patients immediately on arrival in the trauma center; characteristics of these patients are reported in the *SI Appendix*, Table S1. Using unsupervised hierarchical clustering of these variables, we identified 4 high-level clusters of patients based on variations in platelet function that had distinct clinical characteristics and outcomes (Fig. 1 *A* and *B*). In clusters C3 and C4, which contained the most severely injured patients and the highest rates of trauma-induced coagulopathy (TIC), platelet aggregation in response to stimulation with multiple agonists was reduced but thrombin generation was profoundly elevated (Fig. 1*C*). This signature alteration in platelet activity was associated with higher blood transfusion requirements, more than twice the incidence of multiple-organ dysfunction, and up to ten times higher mortality (*SI Appendix*, Table S1). Together these data present a paradoxical situation, where critically bleeding patients lose platelet aggregatory function but maintain the ability to generate thrombin.

### Trauma Patients Develop Procoagulant Balloon Platelets Early in Severe Hemorrhage.

**Fig. 2.**
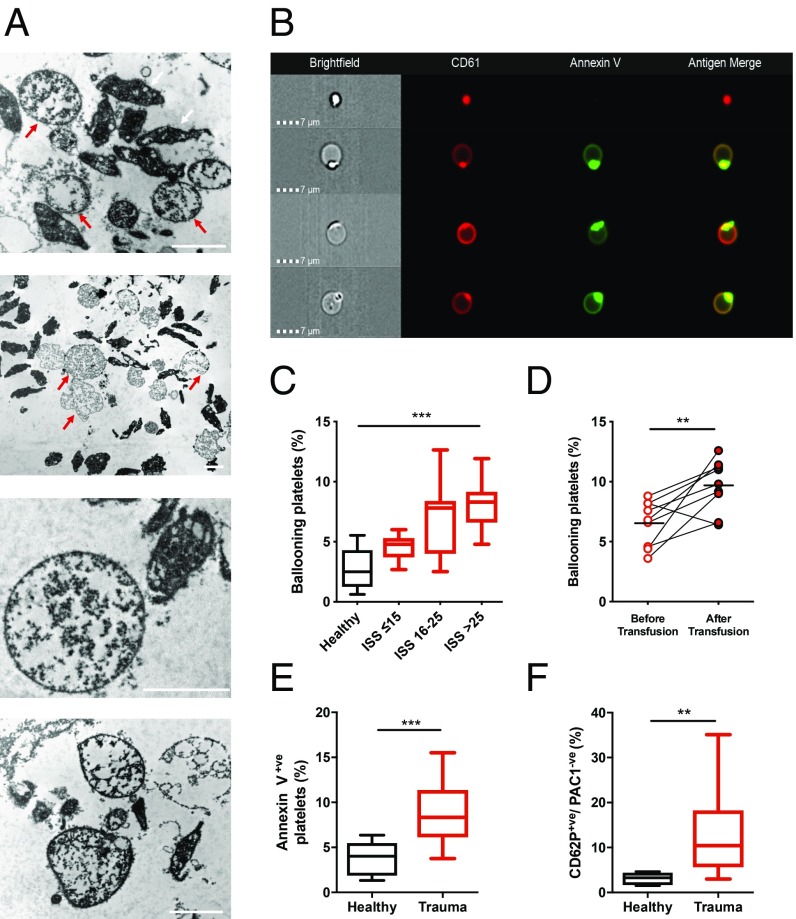
Platelet ballooning during trauma hemorrhage. (*A*) Representative transmission electron microscopy images of platelet-rich plasma from 3 patients with severe injuries showing ballooning platelets (red arrows) with loss of membrane integrity and absent cytoplasmic contents. White arrows indicate nonballooned platelets. (Scale bars, 2 µm.) (*B*) Representative images from trauma patients demonstrating a morphologically normal platelet (*Top*) and platelets displaying membrane ballooning and annexin V binding. (*C*) Number of ballooning platelets in healthy volunteers (black, *n* = 10) and in trauma patients (red) with mild–moderate injuries (ISS ≤ 15, *n* = 13), severe injuries (ISS 16–25, *n* = 17), and critical injuries (ISS > 25, *n* = 18). Results expressed as proportion of platelets. Box plots display median with interquartile range and 10th–90th percentiles. ***P < 0.01, 1-way ANOVA. (*D*) Level of ballooning before (pre-) and after (post-) platelet transfusion in serial samples taken during active hemorrhage (*n* = 9). Lines indicate mean. **P < 0.01, paired *t* test. (*E*) Annexin V binding to platelets isolated from healthy volunteers (*n* = 8) and trauma patients (*n* = 18). ***P < 0.001, Mann–Whitney *U* test. (*F*) Frequency of P-selectin^+ve^/PAC-1^−ve^ platelets in healthy volunteers (*n* = 8) and trauma patients (*n* = 28). **P < 0.01, Mann–Whitney *U* test.

To explore potential mechanisms underlying these observations, we performed a series of experiments on a prospectively recruited cohort of severely injured patients. These patients had clinical characteristics, rates of TIC, and an incidence of platelet dysfunction comparable to those in clusters C3 and C4 (*SI Appendix*, Table S2). We first performed transmission electron microscopy to evaluate changes in platelet morphology. Unexpectedly, we identified large numbers of balloon structures that accumulated during resuscitation and ongoing bleeding (Fig. 2*A*). These structures displayed loss of membrane integrity and absent cytoplasmic contents and were not present in healthy volunteers. We confirmed with imaging flow cytometry that the balloon structures were derived from platelets as they expressed the platelet-specific integrin α_IIb_β_3_ and were procoagulant by annexin V binding in keeping with previous descriptions of platelet balloons (Fig. 2*B* and refs. 13 and 14). The proportion of balloon platelets in the circulation increased as injury severity increased (Fig. 2*C*) and after administration of platelet transfusions (Fig. 2*D*).

Platelet balloons have never been identified in human blood samples ex vivo, but have been generated in vitro and characterized as highly procoagulant (15). The ballooned structures provide a large surface area of phosphatidylserine (PS) on the outer membrane leaflet which enables assembly of the procoagulant enzyme complexes required for thrombin generation (13). In resting platelets from trauma patients on admission, the PS-expressing subset was significantly expanded compared to healthy controls (8.9 ± 1.5% vs. 3.9 ± 1.6%, *P* < 0.001; Fig. 2*E* and *SI Appendix*, Fig. S1). Procoagulant platelets have also been identified as a subpopulation of activated platelets which do not bind PAC-1, a monoclonal antibody targeting the ligand-binding site on the activated conformation of integrin α_IIb_β_3_ (16). The trauma patients had a substantial population of P-selectin–positive/PAC-1–negative platelets, representing a greatly expanded platelet population when compared to healthy volunteers (14.5 ± 5.5% vs. 3.1 ± 1.5%, *P* = 0.004; Fig. 2*F* and *SI Appendix*, Fig. S1). Together, these data indicate a procoagulant ballooning process in trauma patients which had not been identified in human disease and occurs in proportion to the severity of injury.

### Balloon Formation Is Associated with Release of Platelet-Derived Microparticles Which Coat Circulating Leukocytes.

**Fig. 3.**
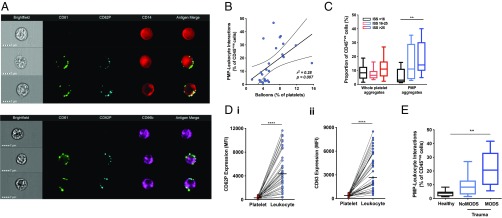
Microparticles released from platelet balloons interact with circulating leukocytes in trauma patients. (*A*) Representative images from imaging flow cytometry showing interactions between platelet-derived microparticles (CD61, green) expressing the platelet activation marker P-selectin (CD62P, blue) with monocytes (CD14, red) and neutrophils (CD66b, purple). (*B*) Correlation between proportion of ballooning platelets and frequency of interactions between PMPs and leukocytes. Dashed lines indicate 95% confidence intervals. (*C*) Impact of injury severity on frequency of interactions between leukocytes and whole platelets or PMPs in trauma patients (*n* = 45). **P < 0.01, 1-way ANOVA. (*D*) Expression of platelet activation markers CD62P (*i*) and CD63 (*ii*) on platelets and CD42b^+ve^ leukocytes from trauma patients (*n* = 30). ****P < 0.0001, student’s *t* test. MFI, mean fluorescence intensity. (*E*) Frequency of PMP-leukocyte aggregates in healthy volunteers (*n* = 10), trauma patients who developed MODS (*n* = 18), and those who did not develop MODS (*n* = 14). **P < 0.01, 1-way ANOVA.

Balloons are friable structures that eventually disintegrate, leading to a surge in microparticle release (13, 17, 18). Levels of platelet-derived microparticles (PMPs) in plasma are known to be elevated in the acute phase after major injury (19, 20). Using imaging flow cytometry, we found that trauma patients’ leukocytes were coated with PMPs in numbers proportional to the numbers of circulating balloons (Fig. 3 *A* and *B*). The proportion of these PMP-covered leukocytes increased with injury severity, whereas whole-platelet leukocyte interactions were infrequent and did not increase (Fig. 3*C*). Whole platelets were minimally activated whereas PMPs on leukocytes were strongly positive for P-selectin and CD63, indicating that they were derived from activated platelets (Fig. 3*D*). Patients who later developed multiple-organ dysfunction syndrome (MODS) were more severely injured than those who recovered without organ complications [injury severity score (ISS) 39 vs. 19, *P* < 0.001] and had a much higher proportion of PMP-coated leukocytes (22 ± 11% vs. 10 ± 5%, *P* = 0.005; Fig. 3*E*).

We postulated that these alterations in platelet structure and function result from exposure to damage-associated molecular patterns (DAMPs), molecules which are released into the extracellular space by activated, damaged, or necrotic cells after injury and which act as a signal that damage has occurred (21). Histones are archetypal DAMPs that are released from damaged tissues into the circulation in high concentrations after severe trauma (12), affect platelet function (22, 23), and induce cytotoxicity through direct membrane disruption (24). We therefore hypothesized that histones may be responsible for platelet ballooning and microparticle release in acute traumatic coagulopathy. We focused on histone H4 in particular, as this has been shown to have the most pronounced effects on platelets and cell membranes compared to other histones (25, 26).

### Histone H4 Is Cytotoxic to Platelets, Inducing Sustained Cytosolic Calcium Elevation and Reducing Agonist Responses.

**Fig. 4.**
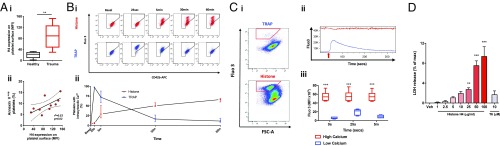
Histone H4 disrupts platelet membrane integrity and results in sustained elevations in cytosolic calcium among a discrete group of platelets. (*A*, *i*) Histone H4 expression on platelets from trauma patients (*n* = 12) and healthy volunteers (*n* = 5). **P < 0.01, student’s *t* test. (*ii*) Association between platelet H4 expression and annexin V–positive platelets. Dashed lines indicate 95% confidence intervals. (*B*) Changes in cytosolic Ca^2+^ in washed platelets from healthy volunteers after stimulation with H4 (red) or TRAP (blue). (*i*) Representative cytometry plots. (*ii*) Median ± interquartile range from 6 independent experiments. (*C*) Responsiveness of washed platelets from healthy volunteers to subsequent stimulation after prolonged H4 exposure. (*i*) Representative cytometry plots after 60-min incubation with H4 or TRAP indicating high-Ca^2+^ (red gate) and low-Ca^2+^ (ungated) populations. (*ii*) Representative kinetic profile of cytosolic Ca^2+^ in the high-Ca^2+^ population (red) and low-Ca^2+^ population (blue) after stimulation with TRAP (red arrow). (*iii*) Trends in platelet cytosolic Ca^2+^ after stimulation with TRAP following preincubation with H4 in high- and low-Ca^2+^ populations. ***P < 0.001 vs. low Ca^2+^ population, 2-way ANOVA with Sidak’s multiple comparisons test, *n* = 6 independent experiments. (*D*) LDH release from platelets after stimulation with H4 or TRAP at the indicated concentrations. Mean ± SEM, *n* ≥ 4 per condition. **P < 0.01 ***P < 0.001 vs. vehicle, 1-way ANOVA with Dunnett’s posttest.

Histone H4 was detectable on the surface of circulating platelets from trauma patients, and the degree of histone binding was strongly correlated with the size of the procoagulant subset (Fig. 4*A*). In vitro, exposure of platelets sourced from healthy volunteers to histone H4 at concentrations previously reported in injured humans produced sustained rises in cytosolic calcium concentrations of platelets which persisted for at least 60 min from exposure (Fig. 4*B*). This population could not respond to subsequent agonist stimulation (Fig. 4*C*). Histone-platelet interaction resulted in membrane damage, evidenced by a concentration-dependent increase in lactate dehydrogenase (LDH) release from platelets (Fig. 4*D*). This was not attenuated by blockade of Toll-like receptor 2 (TLR2) or TLR4, the major receptors for histones on platelets (23) (*SI Appendix*, Fig. S2), consistent with the direct effect that histones are known to exert on cell membranes (24, 27)

### Extracellular Histone H4 Induces Platelet Ballooning and Microparticle Production.

**Fig. 5.**
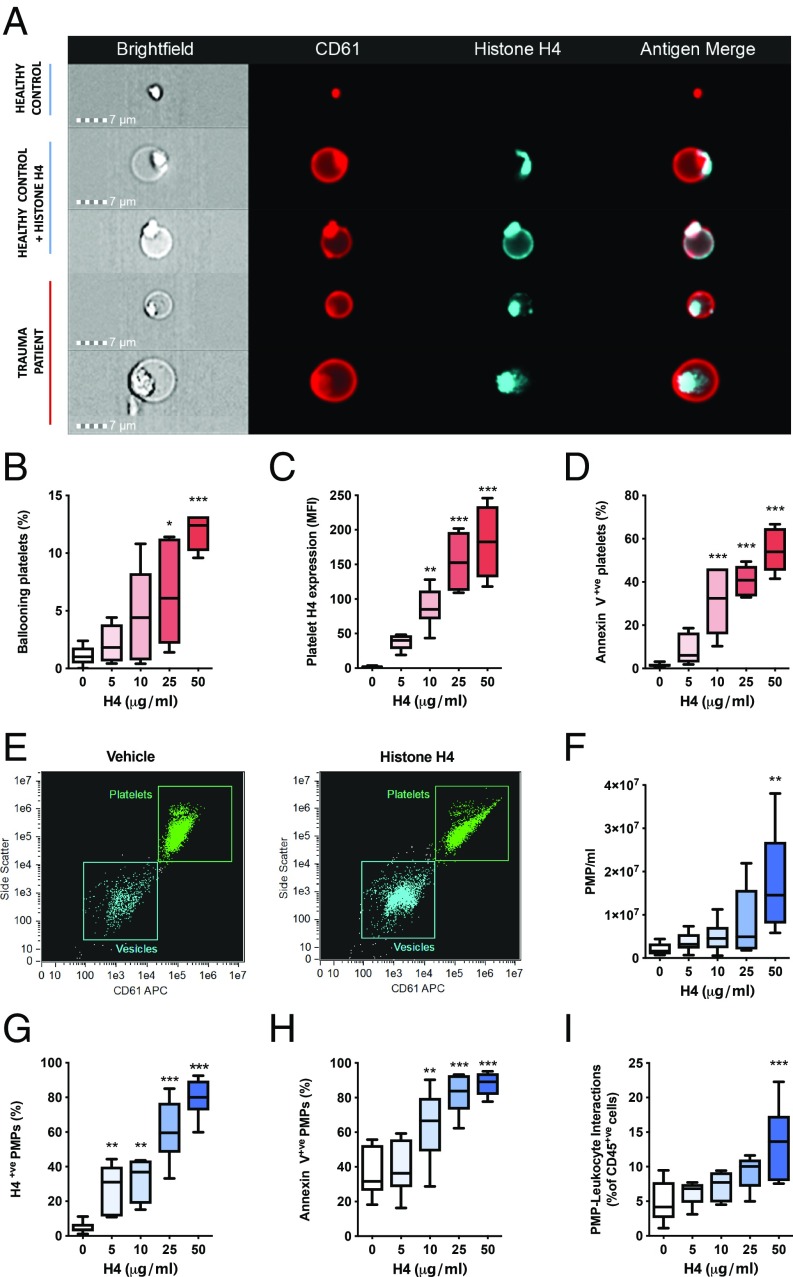
Histone H4 induces platelet ballooning and release of procoagulant, proinflammatory microparticles. (*A*) Representative images of platelets from healthy volunteers and trauma patients. (*B*–*D*) Impact of histone H4 exposure on platelets. Platelet ballooning (*B*), surface expression of histone H4 (*C*), and annexin V binding (*D*) after stimulation with histone H4 at the indicated concentrations or vehicle for 5 min under stirring conditions (1,200 rpm). (*E*–*I*) Histone-induced microparticle production by platelets. Representative flow cytometry plots of vehicle- and histone-treated platelets (*E*). Quantity of PMPs released (*F*), expression of histone H4 on surface of PMPs (*G*), annexin V binding to PMPs (*H*), and interaction of PMPs with leukocytes (*I*). Box plots display median, interquartile range, minimum values, and maximum values from 6 independent experiments. *P < 0.05 **P < 0.01 ***P < 0.001 vs. vehicle, 1-way ANOVA with Dunnett’s posttest.

A sustained elevation in platelet cytosolic calcium is a requirement for balloon formation (13, 28). In accord with this, we found that platelets from healthy individuals exposed to histone H4 were rapidly converted into platelet balloons, with histone deposited both on the remnant body of the ballooning platelets and around the balloon itself (Fig. 5*A*). Histones were also present on the surface of ballooning platelets in samples taken from trauma patients (Fig. 5*A*). Exposure of platelets from healthy volunteers to histone H4 in vitro led to a concentration-dependent increase in expression of histones on the surface of platelets, procoagulant platelet transformation, and platelet ballooning (Fig. 5 *B*–*D*). Blockade of TLR2 or TLR4 in isolation or in combination did not significantly reduce histone-induced platelet ballooning (*SI Appendix*, Fig. S2). Histone H4–treated platelets also produced large quantities of microparticles which retained histone H4 on their surfaces (Fig. 5 *E*–*G*) and expressed phosphatidylserine on their outer leaflets (Fig. 5*H*), suggesting that they originated from the procoagulant subset and indicating their potential to support coagulation and modulate immune cell function. These microparticles coated the surfaces of leukocytes (Fig. 5*I*), reflecting our observations in trauma patients. Finally, histone H4 caused a concentration-dependent release of the proinflammatory alpha granule protein platelet factor 4 (PF4) from platelets in vitro, mirroring observations of elevations in PF4 seen in plasma samples of trauma patients (*SI Appendix*, Fig. S3).

## Discussion

The results presented in this study demonstrate a fundamental switch in platelet behavior toward a procoagulant and proinflammatory phenotype at the expense of platelet aggregation, which occurs during traumatic hemorrhage as a direct response to tissue damage. We propose a model in which histone H4 released into the circulation by mechanically damaged or ischemic tissues exerts a direct cytotoxic effect on platelets. This interaction drives platelet ballooning, leading to the release of microparticles which interact with circulating leukocytes.

Platelet balloons are thought to form at wound sites as a response to vascular injury (13, 28–30). The agonist requirements for platelet ballooning in vitro are high concentrations of collagen (or collagen-related peptide) and thrombin—conditions found at the site of endothelial damage (31). The ballooned platelet membrane maximizes the surface area for assembly of procoagulant enzyme complexes on the platelet surface, which is critical for amplification of thrombin generation at sites of injury (13, 32). Histones have been shown to promote platelet-dependent thrombin generation (23), but have not been shown to induce ballooning. In this study, we observed interactions between histone H4 and platelets in vivo after major injury in humans, and found that this interaction induces sustained rises in cytosolic calcium levels leading to membrane ballooning and procoagulant transformation. Histone H4 is released from tissues which have been mechanically disrupted or subjected to ischemia, resulting in massive elevations in circulating histone levels after severe injury and hemorrhage (12, 33). Of the 5 proteins which make up the histone family, H4 has the most potent effects on platelets (22) and has pore-forming activity on contact with cell membranes which induces lytic cell death (26). Our data suggest that this direct membrane-toxic effect of H4 drives platelet ballooning, although other histones may also play a contributory role. This alternative mechanism accounts for the presence of balloons free in the peripheral circulation in trauma patients at sites remote from vascular damage, and provides a potential explanation for the widespread development of procoagulant platelets in other diseases which involve histone release but not collagen exposure, such as ischemia-reperfusion injury and sepsis-induced disseminated intravascular coagulation (30, 34, 35).

Due to their lack of cytoskeletal architecture, balloons are delicate structures which readily disintegrate, producing large quantities of microparticles (13, 17). Histone H4 exposure recapitulates this phenomenon. In trauma patients, we found large numbers of circulating leukocytes bound by material from activated platelets which was almost entirely in the form of microparticles rather than whole platelets; these interactions were most frequent in patients with severe injuries who developed MODS. Our observations build on previous reports of increased levels of circulating platelet–derived microparticles in plasma from trauma patients (19, 20, 36) and support previous findings suggesting that platelet responses can bridge coagulation and inflammatory systems to shape the immune response during acute sterile inflammation (37). Platelet-derived microparticles have been shown to have immunomodulatory effects on leukocytes (18, 38, 39), and platelet-leukocyte interactions are implicated in organ dysfunction during sterile inflammation in experimental models (40, 41). We postulate that microparticles released from histone-stimulated platelets are an additional facet of the platelet functional repertoire, allowing them to act as messengers which alert the immune response to tissue injury by interactions with circulating neutrophils and monocytes. The molecular interactions involved in these interactions and their ability to cause immunomodulation in trauma patients warrants future research.

Our findings have important implications for the management of trauma hemorrhage and our understanding of TIC. Several authors have described a state of elevated thrombin–generating potential despite loss of procoagulant clotting factors in severely injured patients (42, 43). This study identifies ballooning platelets as the previously undefined procoagulant factor underlying this apparently paradoxical situation. Our findings illustrate that posttraumatic changes in platelet behavior are more complex than solely the impairment of platelet aggregation that has been described (1, 2). It is unclear how platelet ballooning and PMP release impact global assays of hemostasis, such as thromboelastometry, that are increasingly used to guide resuscitation (44). Although allogeneic platelets are routinely administered to bleeding patients as part of major hemorrhage protocols to support platelet function during TIC, there is uncertainty around their efficacy and mechanism of action (45, 46). Transfused platelets are exposed to the same intravascular conditions as endogenous platelets and are therefore susceptible to histone-induced procoagulant transformation. This provides one potential explanation as to why platelet transfusions do not support aggregation (3, 7) but lead to increases in circulating alpha granule proteins (3) and increases in circulating platelet balloons.

In conclusion, this study describes a dramatic phenotypic change in circulating platelets induced by histone release after major trauma. Our findings provide insights into aspects of platelet behavior previously unrecognized in trauma patients, and broaden the concept of platelet “dysfunction” during coagulopathic hemorrhage. We describe a previously undefined and fundamental component of the innate response to damage, which is manifest by the development of platelet ballooning and microparticle production. These observations have implications for the pathophysiology of trauma-induced coagulopathy and multiple-organ dysfunction, and for the future development of effective platelet therapeutics for critically bleeding patients.

## Materials and Methods

Additional methodological details can be found in the *SI Appendix*.

### Study Design.

Adult trauma patients recruited into the Activation of Coagulation and Inflammation after Trauma (ACIT) study who met criteria for advanced trauma team activation at a single urban major trauma center were included in this study. Inclusion and exclusion criteria have been published previously (3, 5). The study was approved by the London – City and East Research Ethics Committee (reference 07/Q0603/29). In patients who lacked capacity, consent for participation was provided by an independent clinician prior to any study-related activities. Informed consent was then obtained from the patient or next of kin at the earliest opportunity. Blood samples were obtained in the emergency department within 2 hours of injury and processed immediately after collection. Characteristics of the study cohorts are described in the *SI Appendix*, Tables S1 and S2. Healthy volunteers taking no regular medication acted as a control group (reference 07/Q0702/24).

### Transmission Electron Microscopy.

Platelet-rich plasma was fixed in graded buffers, washed, and stored overnight in sodium cacodylate buffer. Samples were dehydrated in a graded ethanol series and then infiltrated with London Resin white resin prior to examination with a JOEL JEM-1230 microscope (JOEL USA). Further details can be found in the *SI Appendix*.

### Flow Cytometry and Imaging Flow Cytometry.

P-selectin (CD62P) expression, integrin α_IIb_β_3_ activation, annexin V binding, and histone H4 were quantified on platelets by flow cytometry using an LSRII flow cytometer (Becton Dickinson). Platelet balloons, platelet-leukocyte interactions, and PMPs were characterized and quantified using the ImageStream^x^ Mk II imaging flow cyometer (Amnis). Antibody panels and gating strategies are described in the *SI Appendix*, Figs. S1 and S4.

### Platelet Stimulation.

Washed platelets (3 × 10^8^/mL) were recalcified to 2 mM and incubated at 37 °C under stirring conditions with vehicle or Histone H4 Human, Recombinant (New England Biolabs), at the stated concentrations. Reactions were stopped by addition of 1:2 acid-citrate-dextrose (5 mM dextrose, 6.8 mM trisodium citrate, 3.8 mM citric acid). Platelets were then prepared for flow cytometry or imaging flow cytometry as described in the *SI Appendix*.

### Calcium Mobilization.

Washed platelets were loaded with Fluo 3-AM (Biotium) for 30 min and then incubated with anti-CD42b-APC for 15 min. Platelets were then diluted 1:10 with Tyrode’s buffer with 2 mM calcium. Basal fluorescence was recorded in unstimulated platelets, and changes were quantified in real time following challenge with thrombin receptor–activating peptide 6 (TRAP) or H4 using the LSRII flow cytometer.

### Data Analysis.

Hierarchical clustering analysis was performed with Morpheus software (Broad Institute). Statistical analyses were performed using Prism v6.0 (GraphPad). A 2-tailed *P* value of <0.05 was considered significant throughout.

## Supplementary Material

Supplementary File
