# Supplementary material for: Histone H4 induces platelet ballooning and microparticle release during trauma hemorrhage
Source: Proc Natl Acad Sci U S A. 2019 Aug 12;116(35):17444–9. doi: 10.1073/pnas.1904978116 (PMC6717295; doi:10.1073/pnas.1904978116)
Supplement: Supplementary File [file pnas.1904978116.sapp.pdf]

SI Appendix

Supplemental Tables

Table S1

	All patients	Cluster 1	Cluster 2	Cluster 3	Cluster 4
Number	279	136	81	47	15
Clinical Characteristics					
Age, years	35 (25-50)	34 (23-48)	34 (24-49)	42 (29-62)†	36 (24-57)
Blunt mechanism, n (%)	214 (77%)	99 (73%)	53 (65%)	47 (100%)††	15 (100%)$^{\#}$
Injury Severity Score	18 (9-29)	17 (9-26)	16 (9-22)	27 (20-36)††	29 (22-38)$^{\#}$
Base Deficit, mmol/L	1.5 (-0.7-5.1)	1.4 (-0.5-4.4)	0.7 (-1.2-4)	1.9 (-0.3-6.6)	4.8 (2.7-8.5)$^{\#}$
sBP, mmHg	129 (114-150)	129 (113-149)	134 (115-149)	126 (112-152)	123 (97-166)
Glasgow Coma Score	14 (10-15)	15 (12-15)	15 (11-15)	10 (6-14)††	6 (3-15)$^{\#}$
Functional Coagulation Profile					
Coagulopathy, n (%)[1]	73 (26%)	18 (13%)	27 (33%)	18 (38%)†	10 (67%)$^{\#\#}$
EXTEM alpha angle, degrees	73 (69-76)	74 (70-77)	72 (66-75)**	71 (68-75)†	64 (48-71)$^{\#\#}$
EXTEM MCF, mm	63 (59-67)	65 (61-69)	62 (58-69)*	61 (57-64)††	55 (43-64)$^{\#\#}$
Platelet component, %[2]	77 (71-82)	76 (70-80)	77 (71-83)	79 (73-83)	86 (70-92)$^{\#}$
Platelet hypofunction, n (%)[3]	117 (42%)	12 (9%)	57 (70%)**	38 (81%)††	10 (67%)$^{\#\#}$
Adenosine diphosphate, AU	869 (558-1107)	1077 (890-1279)	658 (437-890)**	568 (332-745)	746 (365-885)$^{\#\#}$
Arachidonic acid, AU	956 (716-1258)	1260 (1088-1443)	740 (443-886)**	736 (525-899)††	723 (614-822)$^{\#\#}$
Collagen, AU	941 (666-1200)	1175 (1000-1422)	681 (394-858)**	666 (456-903)††	766 (473—1141)$^{\#\#}$
TRAP6, AU	1325 (970-1620)	1598 (1407-1822)	994 (785-1254)**	1008 (737-1243)††	1094 (818-1261)$^{\#\#}$
Platelet count, x10^9/L	224 (191-273)	257 (209-268)	212 (186-246)*	201 (145-252)†	217 (165-238)$^{\#}$
Coagulation factors					
PF 1+2	1348 (494-2778)	1141 (410-2109)	671 (319—1104)	3928 (2804-4989)††	10270 (8983-12000)$^{\#\#}$
Fibrinogen, g/L	2.1 (1.5-2.6)	2.2 (1.7)	2.1 (1.6-2.6)	1.9 (1.4-2.2)†	1.2 (0.7-1.5)$^{\#\#}$
Factor II	87 (74-99)	91 (78-103)	89 (77-98)	83 (68-92)†	75 (54-85)$^{\#\#}$
Factor V	82 (61-101)	78 (65-92)	71 (56-89)	71 (56-89)	36 (31-74)$^{\#\#}$
Factor VII	84 (68-101)	86 (68-101)	84 (69-104)	84 (71-102)	76 (57-104)
Factor VIII	294 (208-375)	314 (228-389)	280 (192-356)	250 (184-324)	250 (152-362)
Factor IX	111 (88-133)	114 (93-133)	108 (-80-130)	113 (79-134)	99 (74-134)
Factor X	84 (69-97)	89 (73-99)	85 (68-95)	81 (68-95)	78 (52-96)$^{\#}$
Outcomes					
28-day mortality, n (%)	28 (10%)	6 (4%)	6 (7%)	9 (19%)†	7 (47%)$^{\#\#}$
Major haemorrhage, n (%)[4]	63 (23%)	28 (21%)	12 (15%)	14 (30%)	9 (60%)$^{\#}$
MODS, n (%)[5]	99 (35%)	40 (29%)	17 (21%)	31 (66%)††	11 (73%)$^{\#\#}$
Ventilator days	0 (0-2)	0 (0-1)	0 (0-0)	2 (0-8)††	2 (1-4)$^{\#}$
ICU length of stay, days[6]	0 (0-4)	0 (0-3)	0 (0-0)*	4 (0-11)†	5 (3-17)$^{\#}$
Hospital length of stay, days[6]	12 (4-28)	12 (3-25)	8 (3-15)*	23 (11-47)†	32 (16-52)$^{\#}$

[1]EXTEM CA5 <40mm
[2]Calculated as (EXTEM MCF-FIBTEM MCF)/EXTEM MCF*100
[3]Reduced response to at least one agonist on Multiplate impedance aggregometry
[4]Administration of ≥4 red blood cell units in 24 hours
[5]Multiple organ dysfuntion syndrome; defined as SOFA score ≥6 on at least 2 consecutive days
[6]Survivors only

Table S1: Patient and Laboratory Characteristics by Cluster Grouping. All values are median and interquartile range unless specified. *$p<0.05$ **$p<0.001$, Cluster 2 vs Cluster 1; †$p<0.05$ †† $p<0.001$, Cluster 3 vs Cluster 1; $^{\#}$ $p<0.05$ $^{\#\ \#}$ $p<0.001$ cluster 4 vs cluster 1. Kruskal-Wallis test with Dunn's post-test correction for continuous variables, Fisher's exact test with Bonferroni correction for categorical variables. sBP, systolic blood pressure; MCF, maximal clot firmness; TRAP6, Thrombin receptor activating peptide-6; PF1+2, Prothrombin fragments 1+2; MODS, multiple organ dysfunction syndrome; ICU, intensive care unit.

Table S2

	Trauma patients<br>n=82	Healthy volunteers<br>n=10
Patient characteristics		
Age, years	38 (30-55)	28 (21-36)
Male, n (%)	69 (84%)	6 (60%)
Injury to sample time, minutes	104 (81-115)	-
Admission Physiology		
Heart rate, beats/min	107 (80-127)	-
sBP, mmHg	110 (75-132)	-
Lactate, mmol/L	3.7 (2.0-8.1)	-
Base Deficit, mmol/L	7.3 (2.5-14.2)	-
Glasgow Coma Score	10 (3-14)	-
Injury characteristics		
Blunt mechanism, n (%)	65 (79%)	-
Injury Severity Score	29 (20-41)	-
AIS Head & Neck	0 (0-4)	-
AIS Thorax	3 (0-4)	-
AIS Abdomen	0 (0-3))	-
AIS Extremity and Pelvis	2 (0-4)	-
Coagulation and Platelet Function		
Coagulopathy, n (%)[1]	47 (57%)	-
EXTEM alpha angle, degrees	66 (57-73)	-
EXTEM MCF, mm	57 (45-62)	-
Platelet component, n (%)[2]	81 (75-87)	-
Platelet hypofunction, n (%)[3]	28 (88%)	-
Platelet count, $x10^9$/L	195 (153-243)	-
Fluid & Blood products		
Pre-baseline crystalloid, ml	0 (0-250)	-
Pre-baseline PRBC, units	1 (0-3)	-
24hr PRBC, units	4 (1-7)	-
Major hemorrhage, n (%)[4]	45 (55%)	-
Outcomes		
28-day mortality, n (%)	30 (37%)	
Ventilator days	1 (1-8)	-
Vasopressor days	1 (0-4)	-
MODS, n (%)[5]	35 (43%)	-
ICU length of stay, days[6]	8 (2-16)	-
Hospital length of stay, days[6]	21 (10-53)	-

[1] EXTEM CA5 <40mm
[2] Calculated as (EXTEM MCF-FIBTEM MCF)/EXTEM MCF *100
[3] Reduced response to at least one agonist on citrated Multiplate; results are from a subgroup of 32 patients
[4] Administration of ≥4PRBCs in 24 hours
[5] SOFA score ≥6 on at least 2 consecutive days
[6] Survivors only

Table S2: Characteristics of the Experimental Group and Healthy Volunteers. sBP, systolic blood pressure; AIS, abbreviated injury score; MCF, maximal clot firmness; PRBC, packed red blood cells; ICU, intensive care unit; MODS, multiple organ dysfunction syndrome.

Supplemental Figures

Figure S1

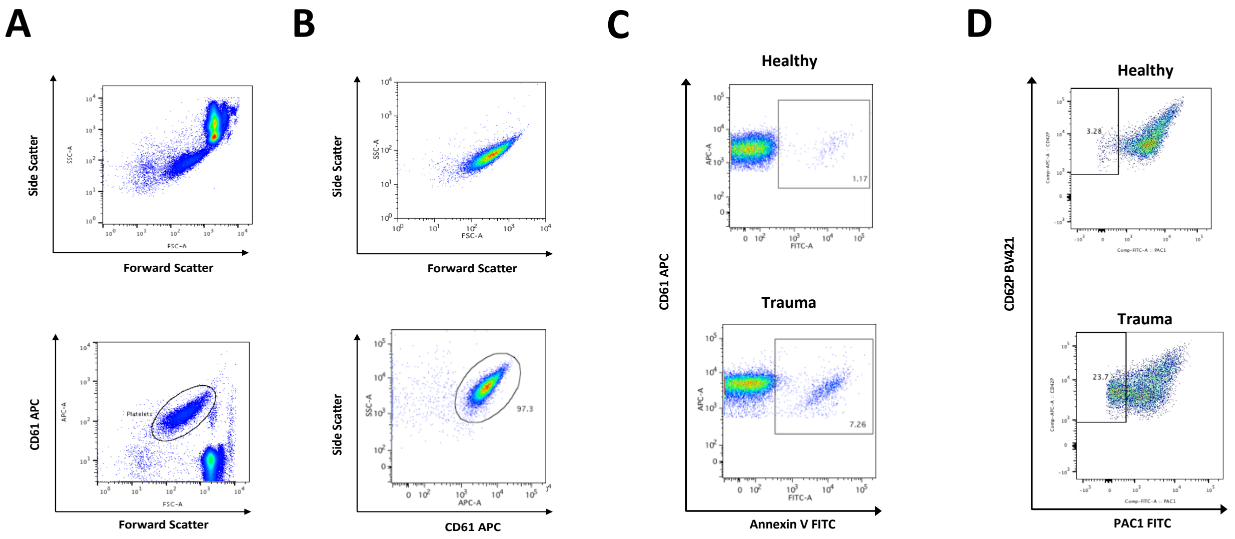

Figure S1: Gating strategy for platelet identification and quantification of the procoagulant subset. A and B: Platelet identification in whole blood (**A**) and washed platelet preparations (**B**) based on forward/side scatter profile and expression of GPIIIa (CD61)**. C and D:** Representative cytometry plots showing Annexin V binding on platelets (**C**) and the frequency of P-selectin^{+ve}/PAC-1^{-ve} platelets (**D**) in healthy volunteers and trauma patients.

Figure S2

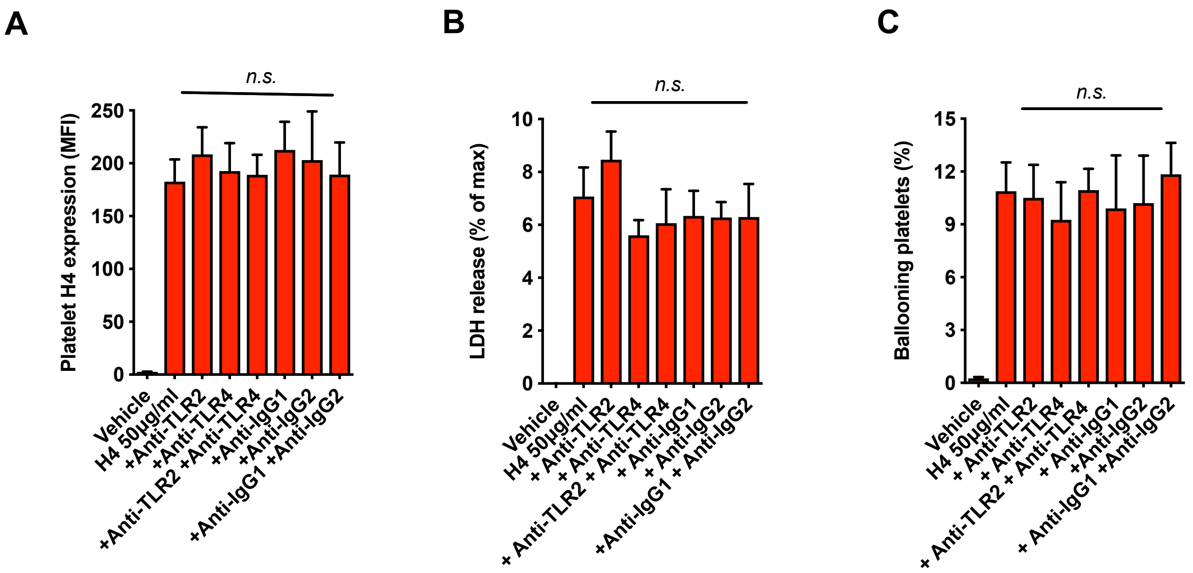

Figure S2: Effect of TLR blockade on histone H4 binding to platelets (A), histone-induced LDH release (B) and histone-induced platelet ballooning (C). Washed platelets were incubated with blocking antibodies to toll-like receptors 2 and/or 4 or class-matched isotype controls prior to stimulation with histone H4 (50µg/ml) for 5 minutes under stirring conditions (1200rpm). Mean ± SEM from ≥4 independent experiments. n.s., not significant vs H4 50µg/ml., one-way ANOVA with Dunnett's post-test for multiple comparisons.

Figure S3

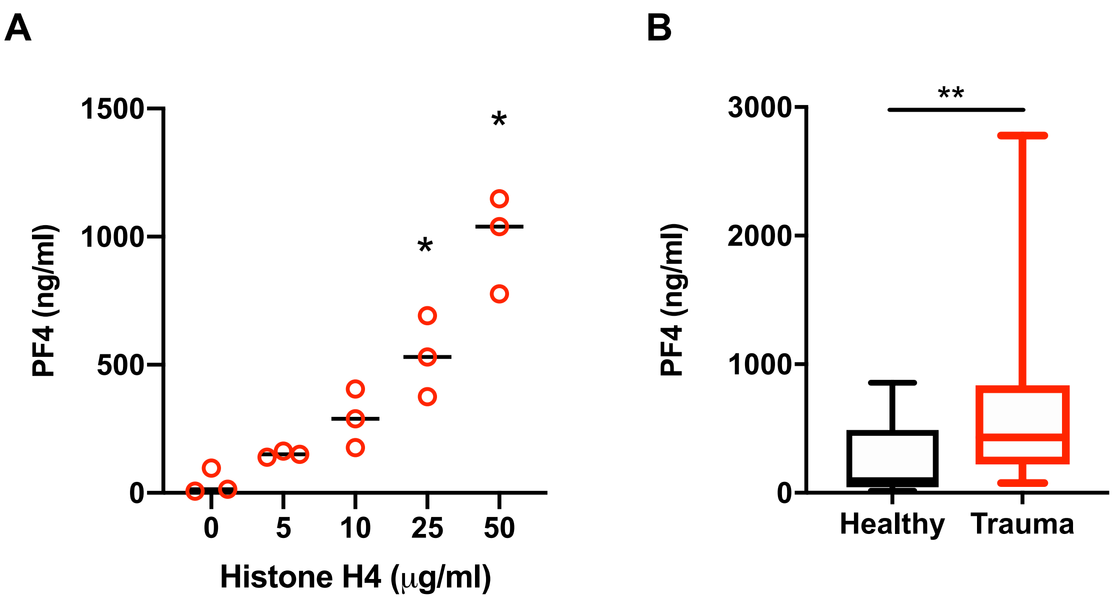

Figure S3: Platelet factor 4 (PF4) release from histone H4-treated platelets (A) and in plasma samples from trauma patients compared to healthy volunteers (B). A: PF4 levels in supernatant from washed platelets after stimulation for 5 minutes with histone H4 under stirring conditions (1200rpm) at the indicated concentrations. *p<0.05 vs vehicle, n=3 independent experiments. **B:** Concentration of PF4 in platelet-poor plasma samples from healthy volunteers (n=15) and trauma patients (n=30). Box plots indicate median, interquartile range and 10-90th percentiles. **p<0.01, Mann-Whitney U-test.

Figure S4

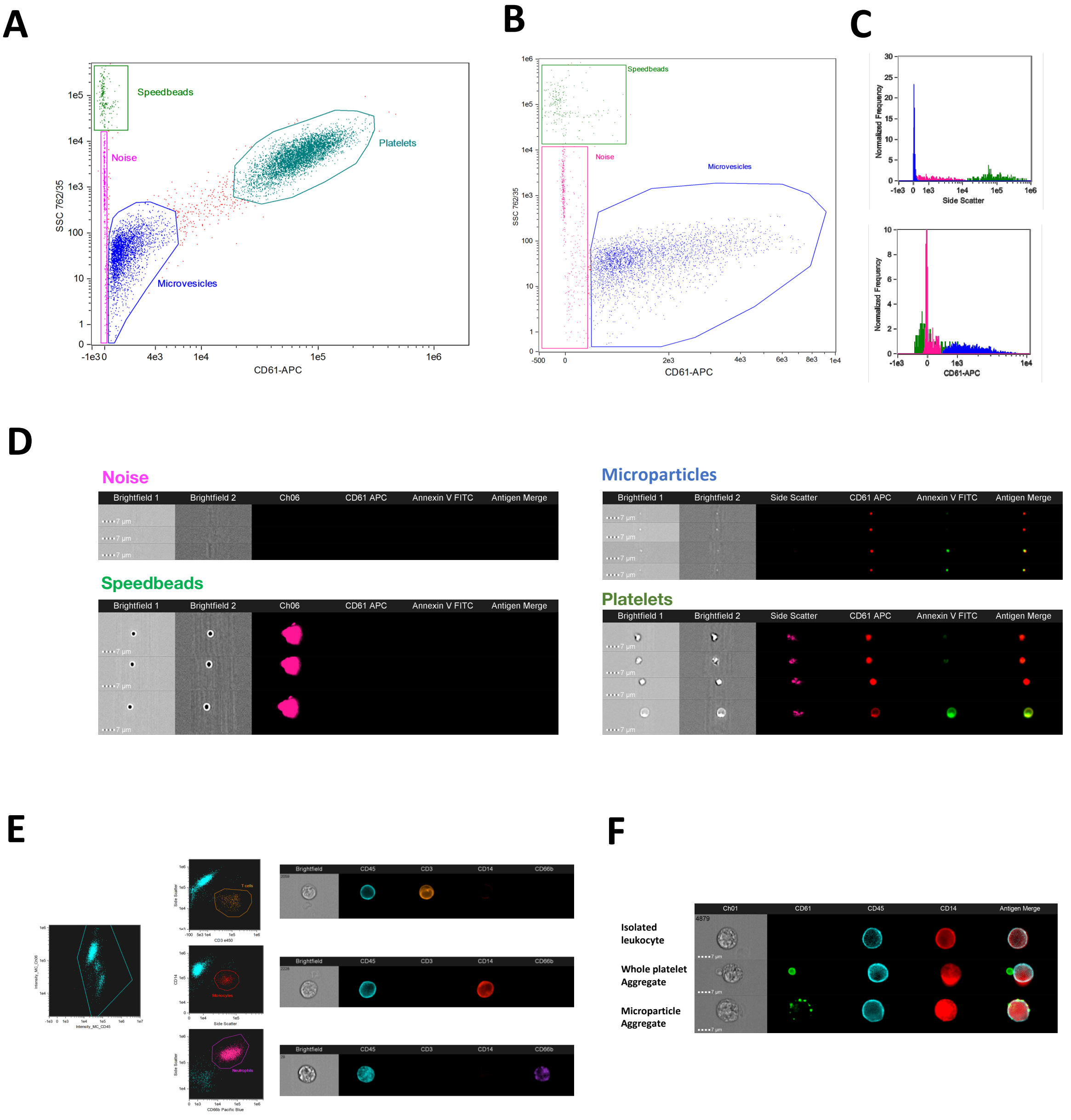

Figure S4: Gating strategy for identification and characterization of platelets and platelet-derived microparticles using flow cytometry. A: Representative scatter plot showing method of discrimination of platelets (dark green) and platelet-derived microparticles (blue) from background noise (pink) and calibration beads (green) using side scatter (y-axis) and CD61 intensity (x axis). **B and C:** Zoomed-in scatter plot (**B**) and histogram profiles (**C**) illustrating discrimination of microparticles from background noise and speedbeads. **D:** Representative microscopy images from the four gates shown in panel A and characterization of platelets and platelet derived microparticles based on morphology and expression of annexin V. **E:** Gating strategy for leukocyte identification using imaging flow cytometry. **F:** Representative images of isolated leukocyte and platelet-leukocyte interactions classified by characteristics.

Supplemental Materials and Methods

Study Design

Adult trauma patients were recruited into the Activation of Coagulation and Inflammation after Trauma (ACIT) study at a single major trauma center. The study was approved by the London (City and East) research ethics committee (reference 07/Q0603/29). Patients meeting criteria for advanced trauma team and/or major hemorrhage protocol activation (systolic blood pressure <90mmHg, poor response to initial fluid resuscitation and/or suspected active hemorrhage) were screened for inclusion. Exclusion criteria were presentation to hospital >2hours from injury, pre-hospital administration of >2000ml crystalloid, burns involving >5% body surface area, known bleeding diathesis and pre-injury use of anticoagulants or antiplatelet agents. Assent for participation was provided by an independent clinician prior to any study related activities, and informed consent from patient or next of kin obtained at the earliest opportunity. Because of the logistical challenges of platelet function testing in this population, separate but overlapping cohorts of patients meeting identical inclusion and exclusion criteria were used for the individual assays. Characteristics of the study cohorts are described in the supplemental material **(Supplemental Tables 1-2)**. Healthy volunteers taking no regular medication acted as a control group (07/Q0702/24).

Study procedures and definitions

Blood was drawn into citrated vacutainers (0.32%, Becton Dickinson (BD), NJ, USA) or hirudin (from healthy volunteers and within 20 minutes of trauma patient arrival in the emergency department. Physiological variables, patient demographics, injury characteristics and medical interventions were recorded in real-time by the recruiting investigator. Patients were followed up on a daily basis from admission until day 28 unless death or hospital discharge occurred before this time. Multiple organ dysfunction syndrome (MODS) was defined as a sequential organ failure assessment score of ≥6 on

≥2 consecutive days, excluding the first 48hours after admission (1, 2). Severe and critical injury were defined as injury severity scores of >15 and >25 respectively (3).

Preparation of platelet rich plasma and washed platelets

Citrated blood was centrifuged at 175 x *g* for 15minutes at room temperature to generate platelet rich plasma (PRP). Washed platelets were prepared by further centrifugation at 1000 x *g* for 10 minutes in the presence of prostacyclin (PGI_2, 2μg/ml, Tocris Bioscience, UK) and apyrase (0.02U/ml, Sigma, UK). The resulting pellet was resuspended in modified Tyrode's buffer containing HEPES (20mmol/L, Sigma) and 0.35% bovine serum albumin (BSA, Sigma) at pH 7.4, centrifuged at 1000 x *g* for a further 10minutes after addition of PGI_2 (2μg/ml), resuspended and diluted to a platelet count of 3 x 10^8/ml.

Platelet aggregometry

Impendence aggregometry was performed with the Multiplate device (Roche Pharmaceuticals, Switzerland) as previously described (4). Briefly, 300μl aliquots of hirudinated (15μg/ml) or citrated whole blood were diluted 1:1 with 0.9% sodium chloride and stimulated with adenosine diphosphate (ADP, 6.5μM), arachidonic acid (AA, 0.5mM), type I collagen (3.2μg/ml) or thrombin-receptor activating peptide-6 (TRAP6, 32μM) under stirring conditions at 37°C. Citrated samples were recalcified to 3mM (final) with $CaCl_2$ prior to stimulation, as per the manufacturer's instructions. Aggregation was then measured over 6 minutes and reported as area under the curve in arbitrary units.

Thromboelastometry

Rotational thromboelastometry (ROTEM) was performed according to the manufacturer's instructions using a ROTEM Delta instrument (TEM International GmBH, Munich, Germany), after addition of tissue factor in the presence (FIBTEM) and absence (EXTEM) of the actin polymerization inhibitor cytochalasin D. The platelet component of clot strength (maximal clot firmness, MCF) was calculated as [(EXTEM MCF – FIBTEM MCF)/(EXTEM MCF)] x 100, as described previously (5).

Quantification of prothrombin fragments

Platelet-poor plasma was generated by double centrifugation of citrated whole blood at 1,700 x *g* for 10 minutes and immediately stored at -80°C. Prothrombin fragments 1+2 were quantified immediately after thawing using a sandwich enzyme-linked immunosorbent assay (Enzygnost® PF 1+2, Siemens Healthcare Diagnostics GmbH, Germany).

Transmission Electron Microscopy

PRP was diluted 1:6 with prefixation solution consisting of freshly prepared 0.1% EM-grade glutaraldehyde (Agar Scientific) in 0.1M sodium cacodylate (Sigma) buffer pH 7.4 and allowed to stand at room temperature for 30-40 minutes. The PRP-prefixation suspension was centrifuged at 800g for 10 minutes and the supernatant decanted. Samples were then incubated in 3% EM-grade glutaraldehyde in 0.1M cacodylate buffer for 2 hours. The fixation solution was decanted and samples were gently washed with 0.1M cacodilate buffer for 5 minutes at room temperature. Platelets were stored overnight at 4°C in a second rinse of the same buffer. Pre-fixed samples were further fixed in 1I aqueous osmium tetroxide in Milli-Q water for 1hr. Samples were then rinsed in 3 15-minute changes of the same water and dehydrated in a graded ethanol series (50%, 70%, 80%, 95%, 100%) at room temperature for 15 mins, followed by two room temperature rinses of 100% ethanol for 20 mins. Samples were then infiltrated with a 1:1 mixture of LR White resin and ethanol, and secondly with a 100% LR White resin for 1 hour each. Samples were then infiltrated overnight in 100% LR white resin. After infiltration samples were dislodged into resin. The resin drop was then transferred to a size 3 Beem capsule filled with a small portion of fresh LR White resin. Additional resin was layered over the platelet film with a pipette, and the blocks allowed to polymerize in an oven maintained at 60°C. Thin sections were cut with a glass knife at a Reichert Ultracut E microtome and collected on uncoated, 300 mesh copper grids. High contrast was obtained by post-staining with saturated aqueous uranyl acetate and lead citrate (6) for 4 min each. The grids were examined in a JOEL JEM-1230 transmission electron microscope at an accelerating potential of 80 kV.

Flow cytometry

P-selectin (CD62P) expression, integrin $\alpha_{IIb}\beta_3$ activation and histone H4 were quantified on the surface of platelets in unstimulated whole blood by flow cytometry. In some experiments, expression profiles were also measured on platelets following activation with TRAP6 (Bachem, Switzerland) or recombinant histone H4 (New England Biolabs, USA) at the indicated concentrations and durations. Aliquots of whole blood were stained with combinations of anti-CD62P-BV421 (clone AK4, BD), BV421-IgG1 (clone X40, BD), anti-CD63-PE-Cy7 (clone H5C6, BD), PE-Cy7-IgG1 (clone MOPC-21, BD), PAC-1-FITC (BD), anti-histone H4-Alexa Fluor 488 (clone 31830, Abcam, UK), anti-IgG1-Alexa Fluor 488 (Abcam), anti-CD42b-APC (clone HIP1, BD), or anti-CD61-APC (clone VI-PL2, eBioscience, UK) for 20minutes in the dark. RGDS blocking peptide (Sigma, 5mg/ml) was used as a negative control for PAC-1 binding. Samples were then diluted 1:50 in phosphate-buffered saline containing 0.1% formalin (Sigma, UK), 0.1% dextrose and 0.2% BSA as previously described (7). Phosphatidylserine exposure was quantified in recalcified washed platelets (2mM $CaCl_2$) with Annexin V-FITC (BD) and reported as the percentage of CD61-APC-positive platelets. All samples were analyzed with an LSRII flow cytometer (BD). Platelets were identified by size, side scatter profile and surface expression of either CD42b or CD61. A minimum of 10,000 platelets were acquired from each sample using FACSDiva acquisition software.

Imaging Flow Cytometry

Platelet morphology

Ballooning platelets were identified based on characteristic morphology, CD61 positivity and annexin-V positivity in either lysed whole blood or washed platelets using the ImageStream^x MkII. Samples were incubated with anti-CD61-APC and anti-histone H4-Alexa Fluor 488, anti-IgG1-Alexa Fluor 488 or Annexin V-FITC for 20 minutes in the dark. 5,000 CD61-positive events were acquired and the files analyzed using IDEAS software (Amnis, Seattle, WA). Balloons were manually counted

by an assessor blinded to the origin of the sample and expressed as a proportion of the total platelet number.

Platelet-leukocyte interactions

Aliquots of whole blood (50µl) were fixed and erythrocytes lysed by dilution (1:30) in Lyse/Fix buffer (BD) immediately after sample collection. Diluted lysed whole blood was centrifuged for 5 minutes at 3,000 x *g*, washed once in PBS, and stained in the dark with the following antibodies and an Fc-receptor blocker (Miltenyi Biotech, UK) for twenty minutes: anti-CD61-fluorescein isothiocyanate (clone VI-PL2, eBioscience); anti-CD62P-phycoerythrin (clone AK4, eBioscience) or anti-IgG1k-phycoerythrin; anti-CD3-eFluor450 (clone UCHT1, eBioscience,); anti-CD45-PerCP Cyanine5.5 (clone HI30, eBioscience); anti-CD66b-Pacific Blue (clone G10F5, Biolegend, San Diego, USA); and CD14-allophycocyanin (clone 6ID3, eBioscience). Samples were processed using the ImageStream^{X} MkII (Amnis, WA, USA) with 60x magnification at low flow rate. Images and cytometry plots were analyzed by an assessor blinded to the origin of the sample. CD61-positive leukocytes ($CD45^{+}CD61^{+}$ events) were subdivided into coincident events, whole platelet-leukocyte interaction or platelet-derived microparticle-leukocyte aggregates based on the size of CD61 positive material on brightfield and fluorescence imaging (**Supplemental Figure 4**). Results are expressed as a proportion of total leukocytes.

Platelet-derived microparticles

Platelet-derived microparticles were identified in washed platelet preparations with the ImageStream^{X} MkII using a modification of a previously published technique (8). Microparticles were distinguished from whole platelets and speed beads by their distinct forward and side scatter properties, and from debris by surface CD61 expression **(Supplemental Figure 4).**

Platelet stimulation

Washed platelets (3 x 10^8/ml) from healthy volunteers were prepared as described and recalcified to 2mM with $CaCl_2$ immediately prior to stimulation. In some experiments, platelets were incubated at room temperature for 20 minutes with blocking antibodies towards toll-like receptor (TLR) 2 (clone T2.5, Biolegend), TLR4 (clone HTA125, Biolegend) or isotype controls at 50µg/ml before stimulation. Aliquots were transferred to a 96-well plate containing vehicle or recombinant histone H4 at the stated concentrations and incubated at 37°C under stirring conditions (1200rpm) using a BioShake IQ plate shake (Q Instruments, Jena, Germany). After 5 minutes, aliquots from each well were diluted 1:2 with acid-citrate-dextrose (5 mM dextrose, 6.8 mM trisodium citrate, 3.8 mM citric acid) for analysis of P-selectin and histone H4 expression or annexin V binding buffer (Biolegend) for annexin V binding. Samples were then prepared for flow cytometry as described above. Platelet releasate was generated by centrifugation at 1000 x g for 10 minutes.

Platelet Factor 4 measurement

PF4 (CXCL4) was measured in citrated platelet-poor plasma from trauma patients or healthy volunteers and in supernatants from washed platelet preparations using a sandwich enzyme-linked immunosorbent assay (Abcam).

Calcium mobilization

Washed platelets (3 x 10^8/ml) from healthy volunteers were prepared as described above then incubated with Fluo 3-AM (Biotium, CA, USA), 5µM for 30 minutes at 37°C followed by staining with anti-CD42b-APC in the dark at room temperature for 15 minutes. Platelets were then diluted 1:10 with Tyrode's buffer with 2mM calcium. Basal fluorescence was recorded for 30 seconds, followed by challenge with TRAP 6 (10µM) or histone H4 (25µg/ml). Fluorescence dynamics were then recorded for a further 5 minutes. A further group of samples were exposed to prolonged histone

H4 or TRAP6 (30 and 60 minutes at room temperature). An additional group of samples were exposed to Histone H4 for 60 minutes, followed by subsequent TRAP6 (10µM) challenge and their Ca^{2+} associated fluorescence dynamics were recorded for a further 5 minutes. Samples were analyzed on a BD LSRII flow cytometer using FACSDiva acquisition software. Platelets were identified by forward scatter profile and APC-CD42b surface expression.

Cytotoxicity assay

Lactate dehydrogenase (LDH) release from washed platelets was quantified using a commercially available colorimetric assay (Cell death detection kit Plus, Roche) according to the manufacturer's guidance. All samples were assayed in duplicate.

Data Analysis

Flow cytometry plots, brightfield images and fluorescence microscopy images obtained with the ImageStream^{X} MkII were analyzed in IDEAS software (Amnis, Seattle, WA). Conventional flow cytometry data was analyzed using FlowJo software (v8.1, Tree Star, USA) and values reported as mean fluorescence intensity or percent positivity after accounting for appropriate isotype or fluorescence-minus-one controls.

Hierarchical clustering analysis was performed using Morpheus software (Broad Institute, Cambridge, MA, USA). This approach has been previously used in studies of trauma-induced coagulopathy (9). For inclusion in our clustering algorithm we chose parameters relating to agonist-induced *ex-vivo* platelet aggregation (impedance aggregometry in hirudinated whole blood in response to ADP, AA, collagen and TRAP6), thrombin generation (EXTEM alpha angle (10) and prothrombin fragments 1+2), and platelet count. Data were standardized to a minimally injured reference population (ISS 1-4, BD <2mmol/L) using the formula $(\mu\text{-x})/\sigma$, where μ is the mean value of the reference population, x is the observed value to be standardized and σ is the standard deviation of the reference population. Euclidean distance was then calculated and clustering performed by complete linkage. We selected a

dendrogram cut-off which would yield clusters containing a minimum of 10 patients per cluster to enable meaningful statistical analysis between them.

Statistical analysis was performed using Prism v6.0 (GraphPad, CA). Distributions of continuous data were assessed using normal-quantile plots. Normally distributed data are presented as mean ± SEM and were compared with Student's or paired T-tests, or one-way analysis of variance (ANOVA) with Dunnett's post-test for multiple comparisons. Non-normally distributed data are reported as median with interquartile range and were compared with Mann-Whitney U-test or Kruskal-Wallis test with Dunn's post-test for multiple comparisons. Categorical data were compared with Fisher's exact test with Bonferroni correction as appropriate. A two-tailed p value of <0.05 was considered significant throughout.

References

1. Cabrera CP*, et al*. (2017) Signatures of inflammation and impending multiple organ dysfunction in the hyperacute phase of trauma: A prospective cohort study. *PLoS Med* 14(7):e1002352.
2. Antonelli M*, et al*. (1999) Application of SOFA score to trauma patients. Sequential Organ Failure Assessment. *Intensive Care Med* 25(4):389-394.
3. Baker SP, O'Neill B, Haddon W, Jr., & Long WB (1974) The injury severity score: a method for describing patients with multiple injuries and evaluating emergency care. *J Trauma* 14(3):187-196.
4. Vulliamy P*, et al*. (2017) Platelet transfusions reduce fibrinolysis but do not restore platelet function during trauma hemorrhage. *J Trauma Acute Care Surg* 83(3):388-397.
5. Bochsen L, Wiinberg B, Kjelgaard-Hansen M, Steinbruchel DA, & Johansson PI (2007) Evaluation of the TEG platelet mapping assay in blood donors. *Thromb J* 5:3.
6. Reynolds ES (1963) The use of lead citrate at high pH as an electron-opaque stain in electron microscopy. *The Journal of cell biology* 17:208-212.
7. Armstrong PC*, et al*. (2015) Novel whole blood assay for phenotyping platelet reactivity in mice identifies ICAM-1 as a mediator of platelet-monocyte interaction. *Blood* 126(10):e11-18.
8. Headland SE, Jones HR, D'Sa AS, Perretti M, & Norling LV (2014) Cutting-edge analysis of extracellular microparticles using ImageStream(X) imaging flow cytometry. *Sci Rep* 4:5237.
9. White NJ*, et al*. (2015) Early hemostatic responses to trauma identified with hierarchical clustering analysis. *J Thromb Haemost* 13(6):978-988.
10. Schneider T, Siegemund T, Siegemund R, & Petros S (2015) Thrombin generation and rotational thromboelastometry in the healthy adult population. *Hamostaseologie* 35(2):181-186.
